# Tumor-Derived Autophagosomes (DRibbles) Activate Human B Cells to Induce Efficient Antigen-Specific Human Memory T-Cell Responses

**DOI:** 10.3389/fimmu.2021.675822

**Published:** 2021-05-26

**Authors:** Hongyan Ren, Tianyu Zhang, Yongren Wang, Qi Yao, Ziyu Wang, Luyao Zhang, Lixin Wang

**Affiliations:** ^1^ Department of Pathology and Pathophysiology, School of Medicine & Holistic Integrative Medicine, Nanjing University of Chinese Medicine, Nanjing, China; ^2^ Department of Microbiology and Immunology, Medical School, Southeast University, Nanjing, China; ^3^ Department of Hematology and Oncology, Children’s Hospital of Nanjing Medical University, Nanjing, China

**Keywords:** DRibbles, human B cells, activation, cross-presentation, antigen-specific memory T cells

## Abstract

We have reported that tumor-derived autophagosomes (DRibbles) were efficient carriers of tumor antigens and DRibbles antigens could be present by DRibbles-activated B cells to stimulate effect and naïve T cells in mice. However, the effect of DRibbles on human B cells remains unclear. Herein, we found that DRibbles can also efficiently induce proliferation and activation of human B cells and lead to the production of chemokines, cytokines and hematopoietic growth factors. We further demonstrated human B cells can effectively phagocytose DRibbles directly and cross-present DRibbles antigens to stimulate antigen-specific memory T cells. Furthermore, we found that membrane-bound high-mobility group B1 (HMGB1) on DRibbles was crucial for inducing human B cells activation. Therefore, these findings provide further evidence to promote the clinical application of B-DRibbles vaccines.

## Introduction

Autophagy is a homeostatic process in which lipids, superfluous organelles, and damaged cytosolic proteins are packaged in double membrane autophagosomes (1), which may be involved in the biogenesis and secretion of extracellular vesicles (EVs) (2, 3). EVs are membrane-encapsulated vesicles released by cells and the vesicles can mediate intercellular communication, modulate antigen presentation and immune activation (4, 5). Upon inhibition of lysosomal/proteasome activity and induction of autophagy, a wide range of cellular antigens, including defective ribosomal products (DRiPs), ubiquitinated short-lived proteins (SLiPs) and long-lived proteins, are sequestered in the special autophagosomes which we termed DRibbles (DRiPs-containing blebs) (6). We have reported that DRibbles are potent antigen-carriers capable of inducing efficient antigen-specific T cell responses *via* mouse dendritic cells (DCs) (6–9). Recently we found they could be cross-presented to activate human memory T cells by elutriated monocytes (10). As we known, B cells are also professional antigen presenting cells (APCs) that display a remarkable ability to stimulate T cells when activated. What’s more, compared with DCs, it is easier to isolate and amplify B cells from non-stem cell sources *in vitro* (11–13). Therefore, our previous studies with mouse tumor models have proved that DRibbles could activate mouse naïve B-cell and DRibbles-contained antigens could be present by the activated B cells to stimulate effector and naïve T cells (14–16). However, the effect of DRibbles on human B cells remains unclear.

In the present study, we investigated whether tumor-released DRibbles could stimulate human B cells activation and human B cells could phagocytose and present DRibbles antigens to induce antigen-specific human memory T-cell responses. Cytomegalovirus (CMV), a ubiquitous β-herpes virus, leads to a lifelong subclinical infection in the majority of people worldwide. Therefore, CMV-specific memory T cells can be obtained from peripheral blood mononuclear cells (PBMCs) of CMV-positive donors. Here, we isolated T cells from PBMCs of CMV^+^ donors to investigate whether CMVpp65^+^DRibbles could be present to stimulate CMVpp65-specific memory T cells by human B cells.

## Materials and Methods

### Preparation of Human PBMCs

According to the Declaration of Helsinki, informed consent was acquired from all donors. PBMCs from leukocyte concentrates of healthy donor or patients with pathologically confirmed liver carcinomas volunteers were separated by Ficoll density gradient centrifugations. The products of leukapheresis were cryopreserved in liquid nitrogen. Ethical approval for the research was granted by the Human Research Ethics Committee of the Affiliated Hospital of Nanjing University of Chinese Medicine (reference number 2019NL-038-03).

### Cell Lines

The tumor cell lines HepG2.2.15 and LT3 were provided by Prof Jian-qiong Zhang. Prof Hong-ming Hu offered UbiLT3 GFP cell line and UbiLT3 pp65 cell line with expression of immunodominant CMV pp65 antigen. Culture of LT3 cell populations was performed in RPMI 1640 containing 10% fetal bovine serum, 2 mM L-glutamine, 100 mg/ml streptomycin, 100 units/ml penicillin, and 1 mM sodium pyruvate.

### Preparation of DRibbles

DRibbles were enriched from the cells described above as published previously (6). The tumor cells were pretreated with 100 nM Bortezomib (Millennium pharmaceuticals, Cambridge, MA), 100 nM Rapamycin (Enzo Life Sciences, Shanghai, China) and 10 mM NH_4_Cl (Enzo Life Sciences, Shanghai, China) for 18–24 h in a 5% CO2 incubator at 37°C. The cells were harvested and centrifuged at 1,000 rpm for 10 min to remove cells and large cell debris, then the supernatant was centrifuged to pellet the DRibbles at 12,000 rpm for 30 min. The DRibbles were washed three times with PBS and Bicinchoninic acid Protein Assay kit (Beyotime Biotechnology, Shanghai, China) was used to quantify the total protein concentration in DRibbles. In some experiments, the tumor cells were pretreated with Rapamycin (100 nM) alone to isolate the autophagosomes.

### Measurement of DRibbles Induced Human B Cells Proliferation and Activation

Human B cells proliferation was evaluated by CFSE (carboxyfluorescein diacetate succinmidyl ester) dye dilution assay *in vitro*. PBMCs were labeled with CFSE (5 μM, Invitrogen, Catalog #C1157) as proposed by the manufacturer. CFSE-labeled PBMCs (1 × 10^6^/ml, 1 ml) were cultured with HepG2.2.15 DRibbles (0, 10, 30, or 100 μg/ml), total cell lysates (30 μg/ml total proteins) or CpG (10 μM) for 3 days, stained with CD19-APC (0.2 μg/ml, BD Bioscience, Catalog #555415) and then analyzed by FACS. FACS analysis was performed by 4-color flow cytometry using BD Calibur and analyzed using the FlowJo software package. CM (complete medium) and CpG were included as the negative and positive controls.

PBMCs (1 × 10^6^/ml, 1 ml) or purified human B cells (1 × 10^6^/ml, 1 ml) from healthy donor or patients with liver carcinomas were co-cultured with HepG2.2.15 DRibbles (0,10, 30, or 100 μg/ml), or CpG (10 μM) for 3 days. Then, the single-cell suspensions were obtained and stained with CD19-APC (0.2 μg/ml, BD Bioscience, Catalog #555415), CD25-PE (0.2 μg/ml, BD Bioscience, Catalog #555432), HLA-DR-PE (0.2 μg/ml, BD Bioscience, Catalog #555812), CD86-PE (0.2 μg/ml, BD Bioscience, Catalog #555658), and CD80-PE (0.2 μg/ml, BD Bioscience, Catalog #557227). Purified human B cells were isolated by Untouched™ human B cells Dynabeads according to the manufacturer’s protocols (Invitrogen, Catalog # 11351D). In all cases, the purity of isolated human B cells was **≥**95% by flow cytometric analysis (**Supplementary Figure 1**). In some experiments, HepG2.2.15 DRibbles (30 μg/ml) were pretreated with anti-HMGB1 mAb (2 μg/ml, sigma, Catalog #H9537) or control isotype (2 μg/ml, mouse IgG, Invitrogen, Catalog #31903) overnight at 4 °C and then used to stimulate purified B cells (1 × 10^6^/ml) for 72 h, the single-cell suspensions were obtained and stained with CD19-APC (0.2 μg/ml, BD Bioscience, Catalog #555415), HLA-DR-PE (0.2 μg/ml, BD Bioscience, Catalog #555812) and CD86-PE (0.2 μg/ml, BD Bioscience, Catalog #555658).

Cytokine and chemokine production by purified human B cells was evaluated by MILLIPLEX MAP human cytokine/chemokine kit (Millipore). Purified human B cells (1 × 10^6^/ml, 1 ml) were stimulated by HepG2.2.15 DRibbles (0, 10, 30, or 100 μg/ml), or CpG (10 μM) for 3 days and then the supernatant was harvested for detection of chemokines (IP-10, MCP1, MIP1α, IL-8), cytokines (TNF-α, IL-6, IL-1α, IL-10), and hematopoietic growth factors (G-CSF, GM-CSF). Besides, the levels of IL-6 and IL-10 in the supernatant were detected by ELISA according to the manufacturer’s protocols (Multi sciences, Hangzhou, China).

### Phagocytosis of DRibbles

HepG2.2.15 DRibbles were labeled with CFSE according to the manufacturer’s protocol (Invitrogen, C1157). Briefly, HepG2.2.15 DRibbles (30 μg/ml) were stained with 5 μM CFSE at 37°C for 10 min, followed by addition of pre-chilled PBS and incubation on ice for 5 min. The pellet was centrifuged at 12,000 rpm for 30 min. Then CFSE-labeled HepG2.2.15 DRibbles were washed three times with PBS to remove excess CFSE. CFSE-labeled HepG2.2.15 DRibbles (30 μg/ml) were cocultured with the purified human B cells (1 × 10^6^/ml, 1 ml) for 6, 12 and 24 h. The percentage of and absolute numbers of CFSE^+^ human B cells were assessed using FACS.

After 12 h of co-culture with CFSE-labeled HepG2.2.15 DRibbles (30 μg/ml), purified human B cells (1 × 10^6^/ml, 1 ml) were washed with ice-cold PBS, fixed in 4% paraformaldehyde, and finally stained with CD19-PE (0.2 μg/ml, BD Bioscience, Catalog #561741). Confocal microscopy (OLYMPUS FV1000) was used to detect phagocytosis of DRibbles by human B cells. For lysosome tracking, some purified human B cells (1 × 10^6^/ml, 1 ml) treated with CFSE-labeled HepG2.2.15 DRibbles (30 μg/ml) were stained with Lyso-Tracker Red (Beyotime, Catalog #C1046) without fixing. Co-localization of DRibbles and lysosome in human B cells was observed under the confocal laser microscope system (OLYMPUS FV1000) and analyzed by the accompanied software (FV10-ASW and JACop).

### Detection of DRibbles Antigens Cross-Presentation by Human B Cells

Purified human CD8^+^ and CD4^+^ T cells from the lymphocytes of CMV^+^ donors were used as antigen-specific memory T cells. Human CD8^+^ and CD4^+^ T cells were enriched using CD8 MicroBeads (Miltenyi Biotec, Catalog #130-045-201) and CD4 MicroBeads (Miltenyi Biotec, Catalog #130-045-101) respectively following the manufacturer’s instructions. Autologous human B cells (5 × 10^6^/ml, 0.2 ml) were purified and co-incubated with CMVpp65^+^DRibbles (30 μg/ml), control GFP^+^DRibbles (30 μg/ml) or pp65 protein (30 μg/ml) at 37 °C for 12 h, and washed with PBS three times. Subsequently, human B cells loaded with DRibbles or pp65 protein were incubated with purified CD8^+^ (5 × 10^6^/ml, 0.2 ml) or CD4^+^ T cells (5 × 10^6^/ml, 0.2 ml) for 12 h. IFN-γ^+^CD8^+^ and IFN-γ^+^CD4^+^ T cells were analyzed using FACS. PBS and pp65 protein were included as the negative and positive controls. In some experiments, CMVpp65^+^ DRibbles (30 μg/ml) pretreated with anti-HMGB1 mAb (2 μg/ml, sigma, Catalog #H9537), or control isotype (2 μg/ml, mouse IgG, Invitrogen, Catalog #31903) overnight at 4°C were co-cultured with purified human B cells (1 × 10^6^/ml, 0.2 ml) and then used to stimulate autologous purified human CD8^+^T (5 × 10^6^/ml, 0.2 ml) or CD4^+^T cells (5 × 10^6^/ml, 0.2 ml) for intracellular IFN-γ staining.

### Statistical Analysis

Data were derived from at least three independent experiments and analyzed using GraphPad Prism 7.0 software. Multiple group comparisons were performed by one-way ANOVA followed by Tukey’s *post hoc* test. Comparisons between two groups were performed using un-paired Student’s t-test. Differences with *P* < 0.05 were considered significant.

## Results

### DRibbles Induced Human B Cells Proliferation

**Figure 1:**
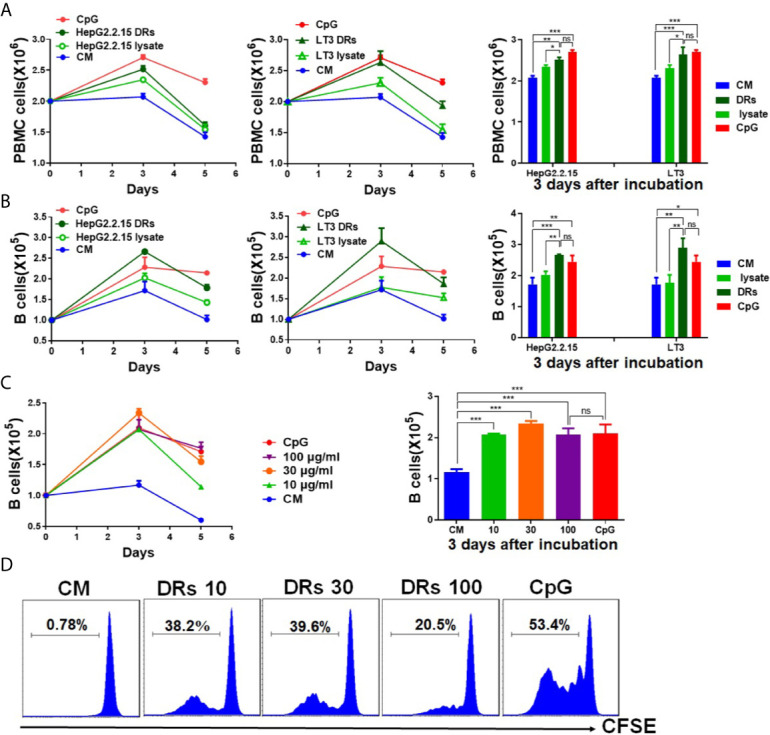
DRibbles induced human B cells proliferation *in vitro*. **(A)** PBMCs isolated from the healthy donors were incubated with DRibbles (30 μg/ml), which were derived from HepG2.2.15 or LT3 cell line, and corresponding tumor cell lysates (30 μg/ml total proteins) for 1 to 5 days. Absolute numbers of PBMCs **(A)** and B cells (CD19^+^cells among PBMCs, **(B)** were measured by flow cytometric analysis. **(C)** PBMCs co-incubated with different concentrations (0,10, 30, or 100 μg/ml) of HepG2.2.15 DRibbles for 3 days, absolute numbers of B cells (CD19^+^cells among PBMCs) were measured by flow cytometric analysis. **(D)** PBMCs were labeled with CFSE and incubated with HepG2.2.15 DRibbles for 3 days, stained with APC-labeled anti-human CD19 mAb and then analyzed by flow cytometry. CM indicated complete medium. Results are representative of three independent experiments from three different healthy donors. ns, *P* > 0.05, *p < 0.05, **p < 0.01, ***p < 0.001.

We have proved that DRibbles had a double-membrane structure and their size was ranged from 100 to 1,000 nm by trans-mission electron microscopy analysis (17). To determine whether DRibbles could stimulate human B cells proliferation, PBMCs prepared from leukocyte concentrates of healthy donor were incubated with DRibbles, which were derived from HepG2.2.15 or LT3 cell line, and corresponding tumor cell lysates for 1 to 5 days. Absolute B cells count in PBMCs were determined by flow cytometry. Compared with PBS or total cell lysates, we found that the number of PBMCs and B cells (CD19^+^cells among PBMCs) increased significantly after incubation with HepG2.2.15 DRibbles (30 μg/ml), LT3 DRibbles (30 μg/ml) or CpG (10 μM, as a positive control), and reached the peak on the 3rd day **(** **Figures 1A, B** **)**. And the promotion effect on human B cells proliferation mediated by DRibbles (30 μg/ml, from HepG2.2.15 or LT3) and CpG (10 μM) appeared to be comparable on the 3rd day **(** **Figures 1A, B** **)**. Moreover, when PBMCs were co-incubated with different concentrations of HepG2.2.15 DRibbles (0,10, 30, or 100 μg/ml) for 3 days, we observed that the optimal concentration of DRibbles was 30 μg/ml, which was sufficient for induction of human B cells proliferation **(** **Figure 1C** **)**. Meanwhile, CFSE-labeled PBMCs were stimulated with HepG2.2.15 DRibbles (0,10, 30, or 100 μg/ml) for 3 days, we found that B cells (CD19^+^cells among PBMCs) was markedly increased similarly **(** **Figure 1D** **)**.

### DRibbles Induced Human B Cells Activation

**Figure 2:**
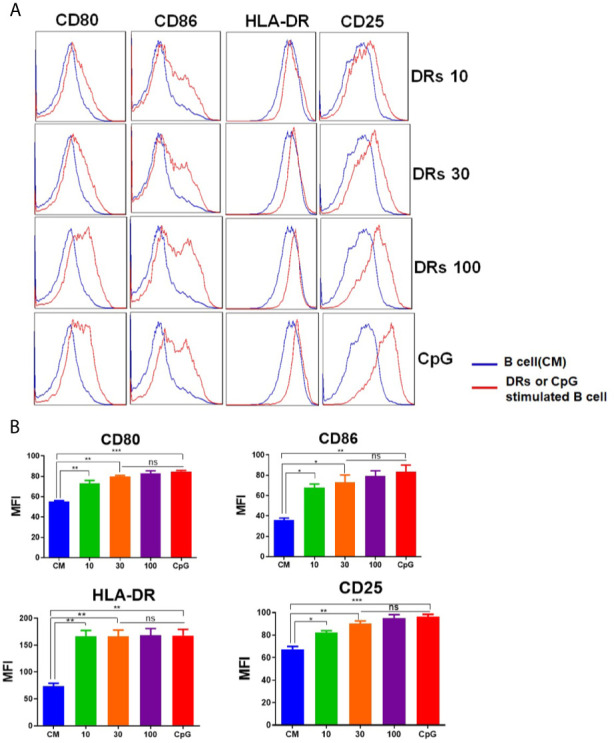
DRibbles-induced activation of human B cells. Purified human B cells were stimulated with various concentrations of HepG2.2.15 DRibbles (0, 10, 30, or 100 μg/ml) or CpG (10 μM). After 72 h incubation, single cell suspensions were collected and measured the expression of costimulatory molecules CD80, CD86, the human major histocompatibility complex (MHC) molecule HLA-DR, and the activation marker CD25 on human B cells by flow cytometric analysis. **(A)** Representative cytoflourograph from one of the donors. **(B)** Cumulative data from three independent experiments from three different healthy donors. CM indicated complete medium. MFI indicated mean fluorescent intensity. ns, *P* > 0.05, *p < 0.05, **p < 0.01, ***p < 0.001.

In order to test the ability of DRibbles to induce human B cells activation, we cultured purified human B cells (97.4% CD19^+^, **Supplementary Figure 1**) with various concentrations of HepG2.2.15 DRibbles (0,10, 30, or 100 μg/ml) or CpG for 3 days and measured the expression of HLA-DR (the human major histocompatibility complex molecule), costimulatory molecules CD80, CD86, and the activation marker CD25 on human B cells by FACS analysis. We found, as expected, DRibbles stimulation significantly increased HLA-DR, CD80, CD86 and CD25 expression on human B cells **(** **Figures 2A, B** **)**. In addition, stimulation with the autophagosomes isolated from HepG2.2.15 pretreated with Rapamycin alone could also significantly increase HLA-DR and CD86 expression on human B cells, but the promotion effect on the activation of human B cells was weaker than that of Bortezomib and Rapamycin pretreatment group (**Supplementary Figure 2A**). And HepG2.2.15 DRibbles stimulation dramatically upregulated the expression of HLA-DR and CD86 on human B cells from patients with liver carcinomas too (**Supplementary Figure 2B**). These data suggest that DRibbles stimulation could efficiently induce the activation of human B cells.

**Figure 3:**
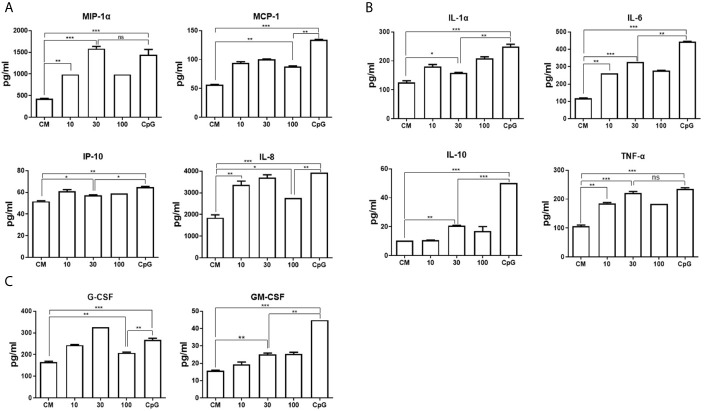
DRibbles-induced chemokines, cytokines and hematopoietic growth factors production by human B cells. The purified human B cells were cocultured with various concentrations of HepG2.2.15 DRibbles (0, 10, 30, or 100 μg/ml) or CpG (10 μM) for 72 h, the supernatants were harvested for examination of chemokines **(A)**, cytokines **(B)** and hematopoietic growth factors **(C)** production by multiplex biomarker immunoassay. CM indicated complete medium. The data represent three separate experiments from three different healthy donors. ns, *P* > 0.05, *p < 0.05, **p < 0.01, ***p < 0.001.

We next investigated whether DRibbles stimulation could induce production of chemokines, cytokines and hematopoietic growth factors by human B cells. The purified human B cells were cocultured with HepG2.2.15 DRibbles (0, 10, 30, or 100 μg/ml) or CpG. Culture supernatants were harvested 72 h later and assessed for secretion of chemokines, cytokine and hematopoietic growth factors by multiplex biomarker immunoassay. It was found that DRibbles stimulation, similar to CpG, lead to significant increase in secretion of chemokines (IL-8, MCP1, MIP1α, IP-10, **Figure 3A**), cytokines (TNF-α, IL-6, IL-1α, IL-10, **Figure 3B**), and growth factors (G-CSF, GM-CSF, **Figure 3C**). The highest secretion of chemokines, cytokines and growth factors by human B cells was observed when the dose of DRibbles was 30 μg/ml, except for IP-10 and IL-1α **(** **Figures 3A, B** **)**. Besides, the data from ELISA analysis confirmed that DRibbles stimulation could significantly increase the levels of IL-6 and IL-10 (**Supplementary Figure 3**). However, we also found that DRibbles stimulation could not induce significant secretion of eotaxin, IL-4 and IL-13 (data not shown), suggesting that priming of B cells by other signal may be required for DRibbles to induce these cytokines secretion, or B cells do not produce these cytokines regardless of any stimulatory signal. These results indicated that DRibbles stimulated the secretion of chemokines, cytokines and hematopoietic growth factors by human B cells.

### Human B Cells Could Take Up DRibbles Directly

**Figure 4:**
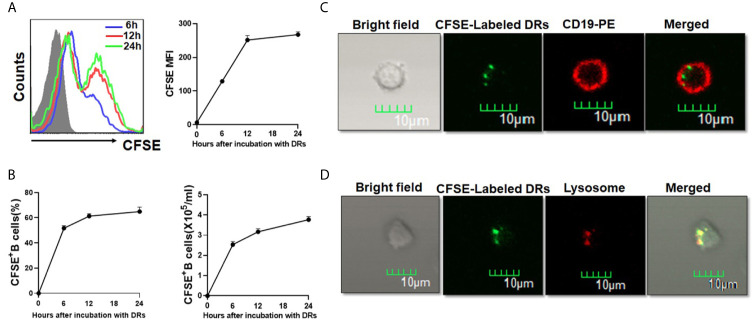
Human B cells could take up DRibbles directly *in vitro*. **(A)** Purified human B cells were cultured with CFSE-labeled HepG2.2.15 DRibbles for 6, 12 and 24 h, CFSE^+^ human B cells were assessed by flow cytometry. **(B)** The percentage and absolute number of CFSE^+^ human B cells (B cells that had phagocytized CFSE-labeled DRibbles) were assessed by flow cytometry. Purified human B cells were co-incubated with CFSE-labeled HepG2.2.15 DRibbles for 12 h, and stained with PE-conjugated anti-CD19 **(C)** or Lyso-Tracker Red **(D)**, the intracellular localization of CFSE-labeled DRibbles was observed under the confocal laser microscope analysis. MFI indicated mean fluorescent intensity. Data presented were obtained as a result of three separate experiments from three different healthy donors.

To test whether human B cells could capture DRibbles directly, HepG2.2.15 DRibbles were labeled with CFSE and cultured with purified human B cells. As shown in **Figure 4A**, the percentage and mean fluorescence intensity (MFI) of CFSE^+^B cells increased after 6 hours of culture, and then increased over time. We found that approximately 60% of human B cells took up DRibbles during incubation for 12 h, and the absolute number of B cells that had phagocytized CFSE-labeled DRibbles increased accordingly (**Figure 4B**). Consistently, immunofluorescence analysis demonstrated that DRibbles labeled with CFSE were internalized by human B cells after 12 h of incubation (**Figure 4C**). Meanwhile, we found that after 12 h of coculture of human B cells with CFSE-DRibbles, the majority of DRibbles colocated with lysosomes (**Figure 4D**), suggesting that DRibbles could fuse with lysosomes after entering into cytosol of human B cells. Collectively, these results demonstrated that human B cells could phagocytize DRibbles rapidly and effectively.

### Human B Cells Cross-Present DRibbles Antigens to Stimulate Antigen Specific Memory T Cells Efficiently

**Figure 5:**
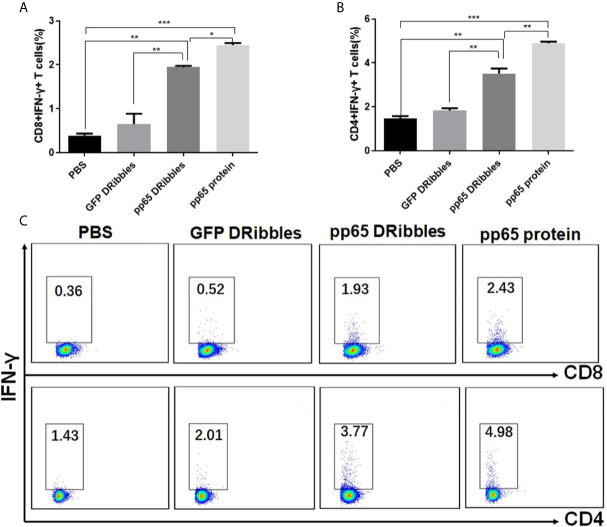
Human B cells could effectively present DRibbles antigens to activate human memory CD8^+^ and CD4^+^ T cells. Purified human B cells from PBMC of CMV positive donors were loaded with CMVpp65^+^DRibbles, control GFP^+^DRibbles or pp65 protein. After 12 h, autologous purified human CD8^+^T or CD4^+^T cells were added. Intracellular cytokine staining (ICS) was employed to calculate the percentage of CD8^+^IFN-γ^+^ T cells **(A)** and CD4^+^IFN-γ^+^ T cells **(B)**. **(C)** The representative dot plot from one of the CMV positive donors was shown. Data are representative of three independent experiments with similar results from three different individuals. *p < 0.05, **p < 0.01, ***p < 0.001.

We have reported that DRibbles-contained antigens could be present to activate effect and naïve T cells by DRibbles-activated B cells in mice (15, 16). To investigate whether DRibbles-loaded human B cells could also effectively activate antigen-specific memory T cells, purified human B cells from PBMCs of CMV positive donors were used as APCs and exposed to CMVpp65^+^ DRibbles, control GFP^+^DRibbles or pp65 protein. Autologous preexisting CMVpp65-specific memory T cells were chosen as the responder cells. Intracellular IFN-γ staining was employed to calculate the frequency of the CMVpp65-specific T cells. We found that pp65^+^DRibbles were presented efficiently by human B cells and both the percentages of CD8^+^IFN-γ^+^ (**Figures 5A, C** **)** and CD4^+^IFN-γ^+^ (**Figures 5B, C** **)** T cells upon pp65^+^DRibbles stimulation were significantly higher than the stimulation with control GFP^+^DRibbles or PBS. These data suggested that human B cells can present DRibbles antigens to activate specific memory T cells efficiently.

### HMGB1 of DRibbles Was Critical for Human B Cell Activation

**Figure 6:**
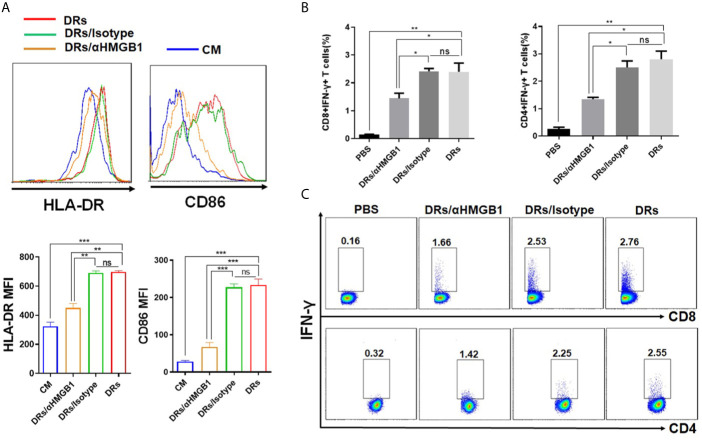
HMGB1 on DRibbles was essential for human B cells activation. **(A)** HepG2.2.15 DRibbles were pretreated with anti-HMGB1 mAb or control isotype and then used to stimulate purified human B cells. After 72 h, the expression of HLA-DR and CD86 on human B cells were assessed by flow cytometry. **(B)** CMVpp65^+^DRibbles pretreated with anti-HMGB1 mAb or control isotype were co-cultured with purified human B cells and then used to stimulate autologous purified human CD8^+^T or CD4^+^T cells. Intracellular cytokine staining (ICS) was employed to calculate the percentage of CD8^+^IFN-γ^+^ T cells and CD4^+^IFN-γ^+^ T cells. **(C)** The representative dot plot from one of the CMV positive donors was shown. MFI indicated mean fluorescent intensity. Data are representative of three independent experiments with similar results from three different individuals. ns, *P* > 0.05, *p < 0.05, **p < 0.01, ***p < 0.001.

HMGB1(the high-mobility group box 1 protein), functions as a damage-associated molecular pattern (DAMP), has been found enriched in and present on the surface of DRibbles (16). And our group has confirmed that HMGB1 was essential for mouse B cell activation (16). Based on these findings, we hypothesized that HMGB1 on DRibbles might play an important role in human B cells activation also. As expected, compared to pre-treatment with isotype antibody, pre-treatment of DRibbles with anti-HMGB1 antibody significantly diminished the upregulation of HLA-DR and CD86 on human B cells (**Figures 6A**), and suppressed IFN-γ secretion by antigen-specific memory CD8^+^T and CD4^+^T cells cocultured with DRibbles-loaded human B cells (**Figures 6B, C** **)**. These results suggested a critical role of HMGB1 in DRibbles-induced human B cells activation and T-cell responses.

## Discussion

We have reported that tumor-derived autophagosomes (DRibbles) were efficient carriers of tumor antigens and multiple antigens encapsulated in DRibbles can be presented to T cells by DCs (6–10). Apart from DCs, we described that B cells from mouse spleen can also be activated by DRibbles (14). However, the effect of DRibbles on human B cells remains unclear. Here, we showed that DRibbles could efficiently induce the proliferation and activation of human B cells. Moreover, we described an effective method to stimulate human memory T-cell responses by activating and inducing human B cells with strong antigen-presenting ability through incubation with DRibbles.

It is widely accepted that the expression of multiple activation markers, such as CD86, CD80 and the major histocompatibility complex, is implicated with the immune activation state of human B cells (18, 19). And it is well known that CD25 is a marker of lymphocyte activation (20). Here, we also found that DRibbles stimulation significantly upregulated the expression of surface CD25, HLA-DR as well as the costimulatory molecules CD80, CD86 on human B cells. The function of B cells as APCs is correlated with the increase in surface expression of the costimulatory molecules. Our data therefore presented novel evidence to support that DRibbles stimulation could lead to human B cells activation and improved antigen presentation. Of note, we found that when the concentration of DRibbles was increased from 30 to 100 μg/ml, the promotion effect on human B cells proliferation decreased, while the effect of DRibbles on phenotypical activation of human B cells was concentration dependent. And our results also showed that the autophagosomes isolated from tumor cells pretreated with the autophagy inducer Rapamycin alone could also induce human B cells activation even in the absence of the proteasome inhibitor Bortezomib. These amazing results and the underlying regulatory mechanisms deserve further investigation in the future.

Activated B cells produce a variety of cytokines, which are involved in B cells maturation and differentiation (20, 21). Recent studies suggested that regulatory B cells regulate the T-cell responses primarily by IL-10 (22, 23). Our data demonstrated that DRibbles stimulation could prominently increase the secretion of pro- and anti-inflammatory cytokines (TNF-α, IL-6, IL-1α, IL-10) by human B cells. In addition, we observed that DRibbles stimulation also enhanced chemokines (IP-10, MCP1, MIP1α, IL-8) and hematopoietic growth factors (G-CSF, GM-CSF) production by human B cells. As we known, MCP1 plays an essential role in DCs migration (24). MIP1α is considered to be crucial for modulating inflammation responses (25, 26), and the expression of MIP1α was increased in patients with chronic myeloid leukemia (27) and acute pancreatitis (28). IP-10 (also called CXCL10) is known as a chemoattractant for T cells activation and influences the generation and trafficking of T cells (29–31). G-CSF and GM-CSF, in addition to regulating the production, differentiation and activation of myeloid cells, also participate in inflammatory responses (32, 33). Our results demonstrated that DRibbles-activated B cells may also have vital roles in regulating the immune responses by secreting the aforementioned chemokines and hematopoietic growth factors. Moreover, mass-spectroscopic analysis of DRibbles we used in this study revealed that DRibbles themselves did not contain the cytokines, chemokines and hematopoietic growth factors mentioned above (data not shown). Further studies are needed to determine whether the cytokines, chemokines and hematopoietic growth factors produced by human B cells could regulate the activity of T cells.

Active internalization by APCs is essential for antigen degradation and presentation to T cells to activate the adaptive immune response. we proved that human B cells could capture DRibbles directly. Moreover, most DRibbles internalized by human B cells were found to co-locate with clustered lysosomes. It was tempting to speculate that DRibbles were finally degraded in lysosomes after entering human B cells, which suggested how to deal with and present DRibbles-contained antigens by activated human B cells.

In the present study, CMVpp65 was chosen as the model antigen to test whether DRibbles antigens could be present by DRibbles-activated human B cells to stimulate memory T-cell responses. DRibbles were enriched from UbiLT3 cells expressing CMVpp65 antigen, which could activate pre-existing pp65-specific human memory T cells  (10). By detecting intracellular IFN-γ production, we showed that human B cells loaded with DRibbles can activate CMVpp65-specific human memory T cells efficiently. Thus, in humans, DRibbles can be presented by B cells to induce efficient T-cell responses.

Our previous studies showed that many DAMP (damage-associated molecular pattern) molecules, such as HSP90 (heat shock protein), HSP94 and HMGB1, were present in DRibbles  (6–8, 16). In this study, we further confirmed that HMGB1on DRibbles was responsible for human B cells activation and subsequent enhanced T-cell responses induced by DRibbles-loaded B Cells. It remains to be determined whether other DAMP molecules, such as HSP90, HSP94 in DRibbles, would play a critical role in human B cells activation in future.

In summary, our studies proved that DRibbles can efficiently activate human B cells and result in the secretion of chemokines, cytokines and hematopoietic growth factors. Furthermore, human B cells could capture DRibbles directly and serve as APCs to stimulate antigen-specific memory T cells. Future studies should further elucidate the mechanisms of human B cells activation induced by DRibbles, which is necessary to foster the clinical development of B-DRibbles vaccine in cancer immunotherapy.

## Data Availability Statement

The original contributions presented in the study are included in the article/**Supplementary Material**. Further inquiries can be directed to the corresponding author.

## Ethics Statement

The studies involving human participants were reviewed and approved by the Human Research Ethics Committee of the Affiliated Hospital of Nanjing University of Chinese Medicine. The patients/participants provided their written informed consent to participate in this study.

## Author Contributions

HR, TZ, YW, and LW designed and performed the experiments. QY, ZW, and LZ provided experimental support. All authors analyzed and discussed the data. HR, TZ, and YW prepared the figures and wrote the manuscript. All authors contributed to the article and approved the submitted version.

## Funding

This work was funded by grants from the National Natural Sciences Foundation of China (81502681, 31970849).

## Conflict of Interest

The authors declare that the research was conducted in the absence of any commercial or financial relationships that could be construed as a potential conflict of interest.

## Supplementary Material

Supplementary Figure 1 Human B cells purified from PBMCs of healthy donor were analyzed by flow cytometry for CD19 expression.

Supplementary Figure 2 The autophagosomes prepared by different pretreatments induced human B cells activation. PBMCs from the healthy donors were stimulated with the autophagosomes (30 μg/ml) isolated from HepG2.2.15 pretreated with Bortezomib and Rapamycin or Rapamycin alone **(A)**, or HepG2.2.15 DRibbles (30 μg/ml) were co-cultured with PBMCs from patients with liver carcinomas **(B)** for 3 days, single cell suspensions were collected and measured the expression of HLA-DR and CD86 on human B cells (CD19^+^) by flow cytometric analysis. CM indicated complete medium. MFI indicated mean fluorescent intensity. Results are representative of three independent experiments from three different donors. **p* < 0.05, ***p* < 0.01, ****p* < 0.001.

Supplementary Figure 3 The secretion of IL-6 and IL-10 by human B cells was measured by ELISA after stimulation with HepG2.2.15 DRibbles (30 μg/ml). CM indicated complete medium. The data represent three separate experiments from three different healthy donors. **p* < 0.05, ***p* < 0.01, ****p* < 0.001.
