# Supplementary figures and images for: Tumor-Derived Autophagosomes (DRibbles) Activate Human B Cells to Induce Efficient Antigen-Specific Human Memory T-Cell Responses
Source: Front Immunol. 2021 May 26;12:675822. doi: 10.3389/fimmu.2021.675822 (PMC8187759; doi:10.3389/fimmu.2021.675822)

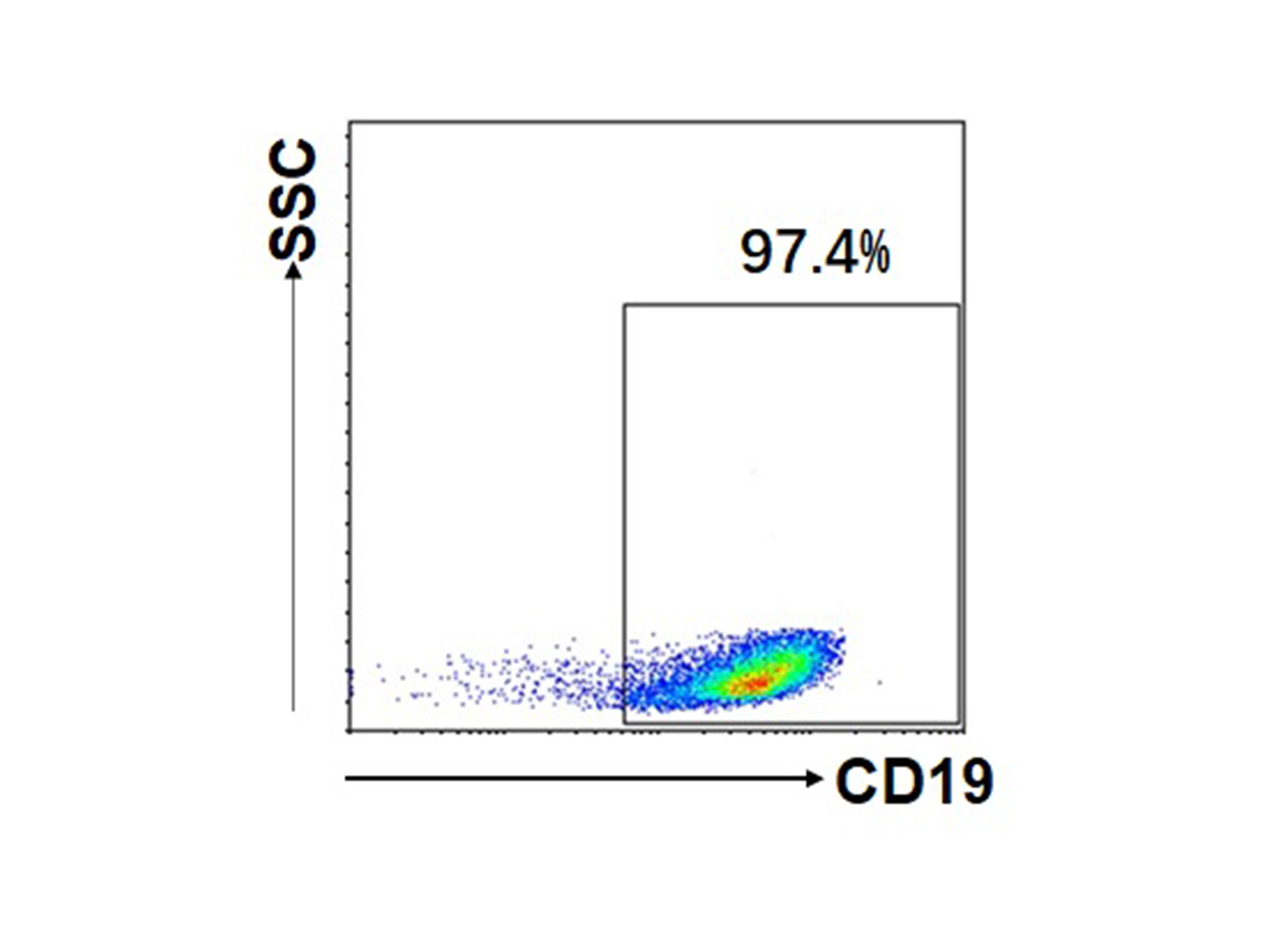

Supplement: Supplementary Figure 1 — Human B cells purified from PBMCs of healthy donor were analyzed by flow cytometry for CD19 expression. [file Image_1.jpeg]

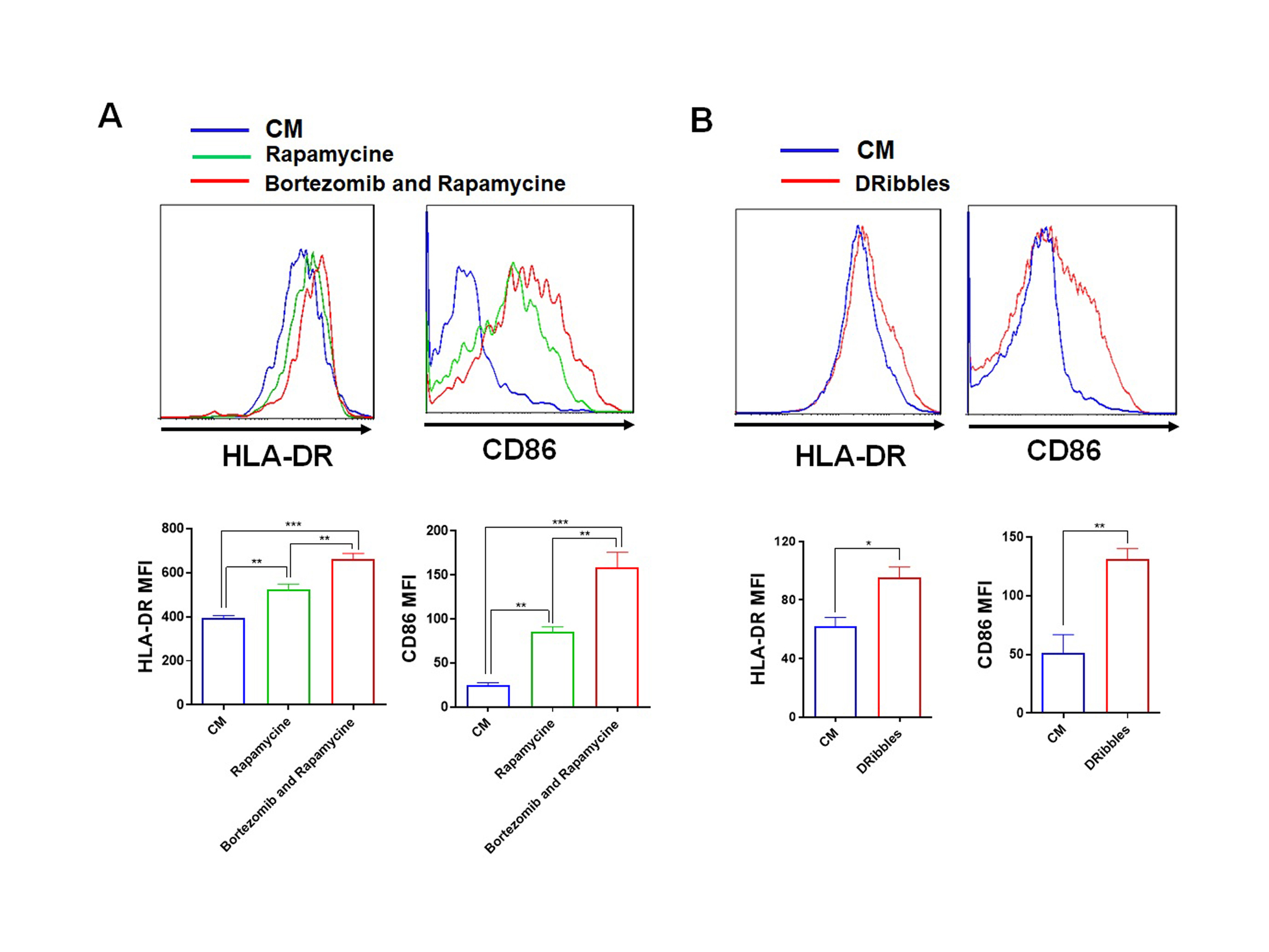

Supplement: Supplementary Figure 2 — The autophagosomes prepared by different pretreatments induced human B cells activation. PBMCs from the healthy donors were stimulated with the autophagosomes (30 μg/ml) isolated from HepG2.2.15 pretreated with Bortezomib and Rapamycin or Rapamycin alone (A), or HepG2.2.15 DRibbles (30 μg/ml) were co-cultured with PBMCs from patients with liver carcinomas (B) for 3 days, single cell suspensions were collected and measured the expression of HLA-DR and CD86 on human B cells (CD19+) by flow cytometric analysis. CM indicated complete medium. MFI indicated mean fluorescent intensity. Results are representative of three independent experiments from three different donors. *p < 0.05, **p < 0.01, ***p < 0.001. [file Image_2.jpeg]

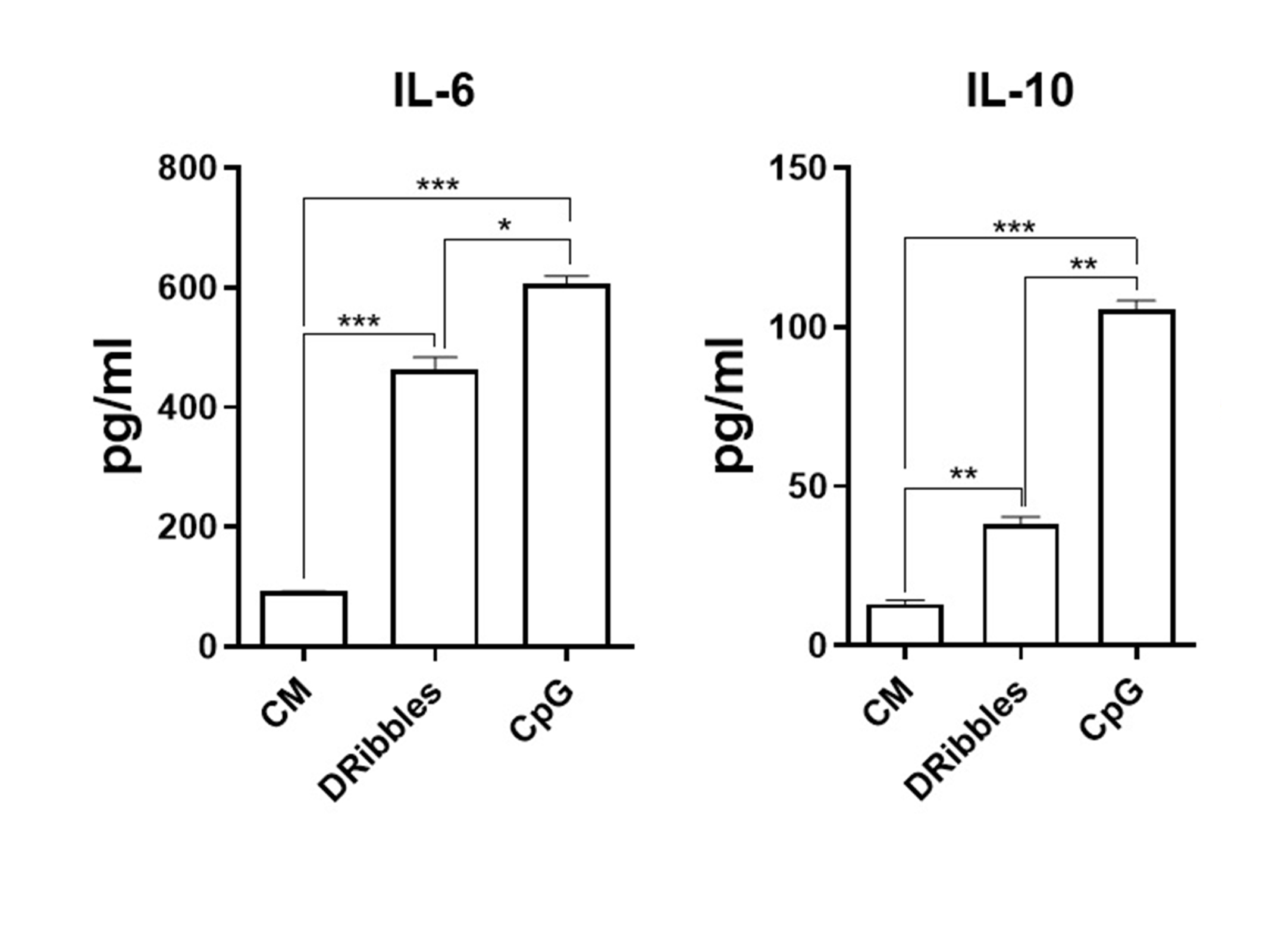

Supplement: Supplementary Figure 3 — The secretion of IL-6 and IL-10 by human B cells was measured by ELISA after stimulation with HepG2.2.15 DRibbles (30 μg/ml). CM indicated complete medium. The data represent three separate experiments from three different healthy donors. *p < 0.05, **p < 0.01, ***p < 0.001. [file Image_3.jpeg]
